# An Anterior Midline Skull Base Epidermoid Cyst Presenting With Spontaneous Intraparenchymal Rupture: A Case Report

**DOI:** 10.7759/cureus.70076

**Published:** 2024-09-24

**Authors:** Lydia Leavitt, Raja Jani, Maunil Mullick, Ramin E Hamidi, Eyas M Hattab, Brian J Williams

**Affiliations:** 1 Neurosurgery, University of Louisville, Louisville, USA; 2 Diagnostic Radiology, University of Louisville, Louisville, USA; 3 Pathology and Laboratory Medicine, University of Louisville, Louisville, USA

**Keywords:** benign intracranial tumor, brain cyst, cranial neurosurgery, epidermoid cysts, mollaret's meningitis

## Abstract

Intracranial epidermoid cysts (ECs) are rare, benign lesions typically found in the cerebellopontine angle, suprasellar spaces, and middle cranial fossa. While these cysts are congenital, originating from ectodermal cell remnants during embryogenesis, it is not until middle age that patients present with symptoms secondary to local mass effect. Here, we present an interesting case of an EC arising from the midline anterior skull base in a 69-year-old male, presenting with symptoms ensuing from the spontaneous rupture of cyst contents into the brain parenchyma. As this is a highly unusual location and presentation for intracranial ECs, this report provides valuable information to the literature on ECs.

## Introduction

Intracranial epidermoid cysts (ECs) are rare, accounting for approximately 0.3-1.8% of all intracranial tumors [1-3]. These cysts are typically congenital in nature, arising from the inclusion of ectodermal elements during neural tube closure in the third to fifth week of gestation [2,4]. Typical locations for these cysts include the cerebellopontine cisterns, suprasellar spaces, and middle cranial fossa [5-7]. Clinical presentation is secondary to the local mass effect. On account of their slow-growing nature, cysts typically do not become symptomatic until the third to fifth decades of life, when patients may present with headaches, cranial nerve defects, seizures, or signs and symptoms of elevated intracranial pressure [3,5]. The standard of care in treating intracranial ECs is surgical excision, with the goal of complete resection of the cyst wall to minimize recurrence [5].

We describe the case of a 69-year-old male (J.M.) who presented with a ruptured frontal intracranial EC. We present the case due to the patient’s advanced age at presentation, atypical location of the cyst, unconventional radiographic features, and spontaneous cyst rupture, which caused chemical meningitis.

## Case presentation

**Figure 1:**
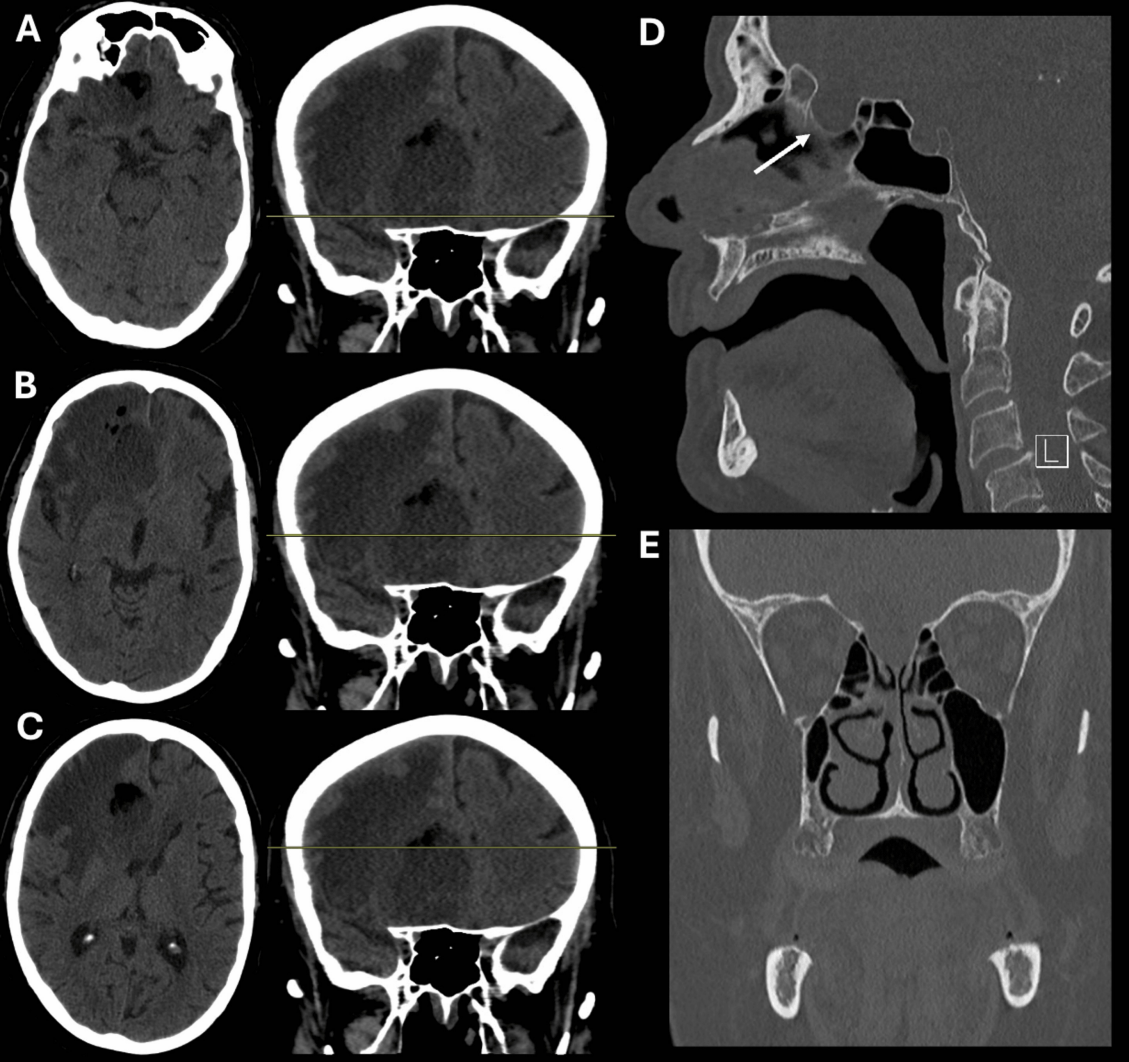
Pre-operative CT scans of head and maxillofacial bones Axial CT head slices, with the level delineated by the yellow line on adjacent coronal images (A-C), demonstrate a mixed-attenuation mass, including fat attenuation (with a CT number of about -29 Hounsfield units), within the inferior medial right frontal lobe, with a large amount of surrounding vasogenic edema and associated local mass effect, resulting in effacement of the anterior aspects of the lateral ventricles (right greater than left) and leftward subfalcine herniation. Sagittal (D) and coronal (E) CT images of the maxillofacial bones demonstrate a well-defined, inferiorly remodeled region of the cribriform plate from just posterior to the crista galli to the planum sphenoidale (white arrow). There is no evidence of dehiscence, destructive bony change, or extension of the tumor into the sinonasal cavity to suggest an aggressive process. CT: Computed tomography

**Figure 2:**
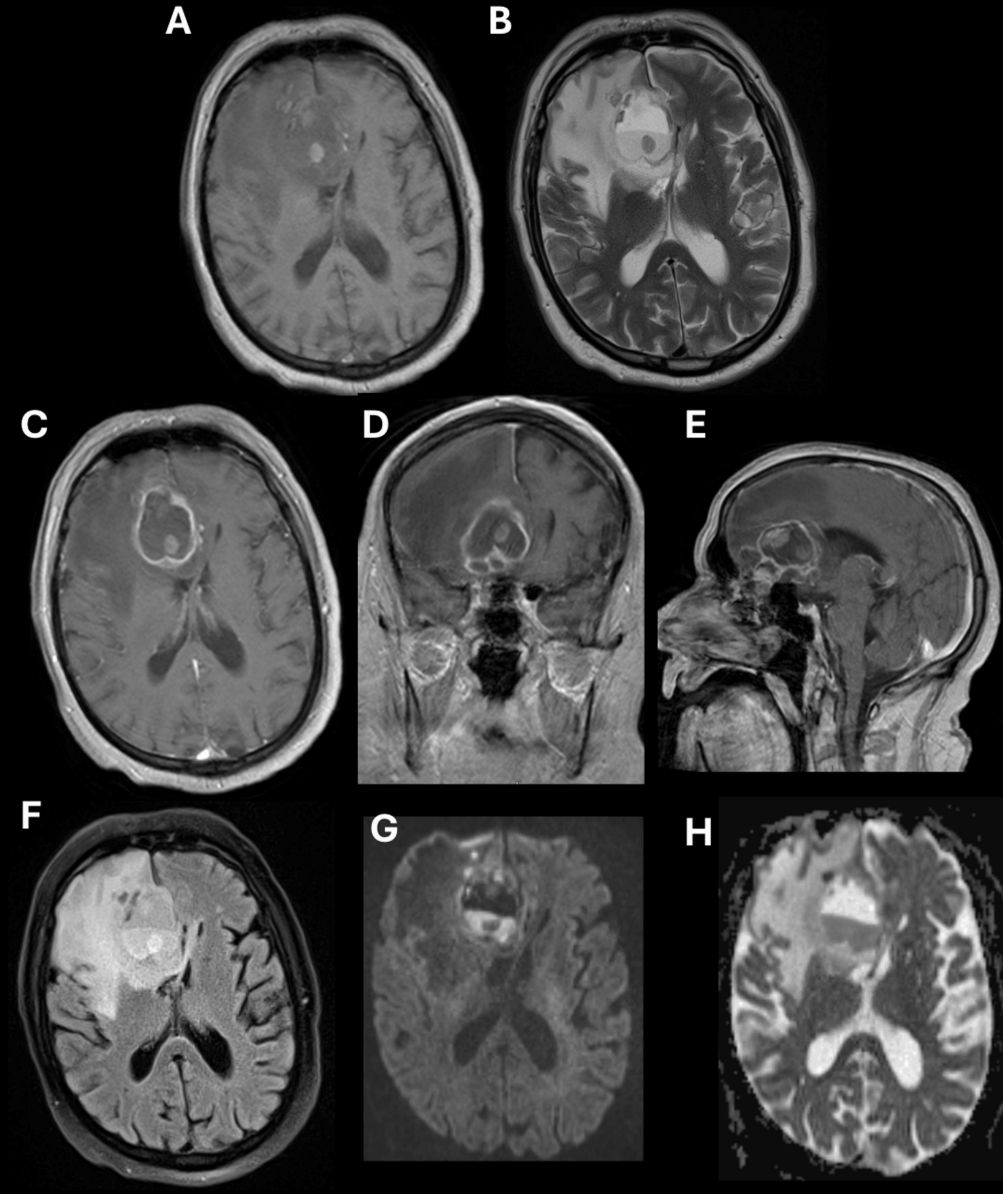
Pre-operative MRI brain MRI of the brain demonstrates a multiloculated, partially cystic, partially nodular mass with some intrinsic T1 hyperintensity (A) and heterogeneous T2 signal within the mass, with marked confluent T2 hyperintensity surrounding the mass (B). The mass demonstrates thin peripheral enhancement on T1 post-contrast images. There is also intrinsic T1 hyperintensity in the mass, suggesting fat or hemorrhagic product (C-E). For example, note the T1 hyperintensity within the mass on pre-contrast T1 images (A), which does not demonstrate enhancement on the post-T1 post-contrast images (C). Vasogenic edema in the right frontal lobe is manifest on axial FLAIR images (F). This is not secondary to infarct or a highly cellular process, as there is no associated diffusion restriction (G) or loss of ADC signal (H). Vasogenic edema is presumed secondary to inflammatory changes resulting from cyst rupture. MRI: Magnetic resonance imaging; FLAIR: Fluid-attenuated inversion recovery; ADC: Apparent diffusion coefficient; CT: Computed tomography

A 69-year-old, right-handed male (J.M.) with a history of heart failure with reduced ejection fraction, atrial fibrillation, hypertension, chronic kidney disease, and nonalcoholic liver cirrhosis presented to the Emergency Department with a one-week history of altered mental status and non-focal weakness, as well as a one-month history of vague headaches. A non-contrast head computed tomography (CT) scan demonstrated an extra-axial, large, mixed-density, non-calcified mass with fat attenuation within the region of the right gyrus rectus. The lesion elevated the undersurface of the right frontal lobe, with effacement of the inferior surface of the anterior horn of the right lateral ventricle. There was extensive surrounding vasogenic edema, which, along with the aforementioned mass, caused a leftward midline shift of approximately 9 mm (Figures 1A-1C). A CT scan of the maxillofacial bones was obtained to further evaluate the relationship of the tumor with the anterior skull base. The CT demonstrated remodeling and enlargement of the cribriform plate, with tumor elements extending into the cribriform plate. There was no evidence of erosive and destructive bony changes or aggressive periosteal reaction, with intact bony margins separating the intracranial compartment from the paranasal sinus compartment (Figures 1D-1E). A magnetic resonance imaging (MRI) scan of the brain, with and without intravenous gadolinium contrast, redemonstrated the anterior skull base right paramedian mass, measuring approximately 3.5 × 5.3 × 4.6 cm, with thin peripheral enhancement. The mass appeared to be predominantly extra-axial and demonstrated diffusion of bright fluid-fluid levels, suggesting blood or proteinaceous material (Figure 2). There were redemonstrations of T2 bright vasogenic edema. Imaging of the chest, abdomen, and pelvis confirmed the absence of underlying systemic malignancy. 

**Figure 3:**
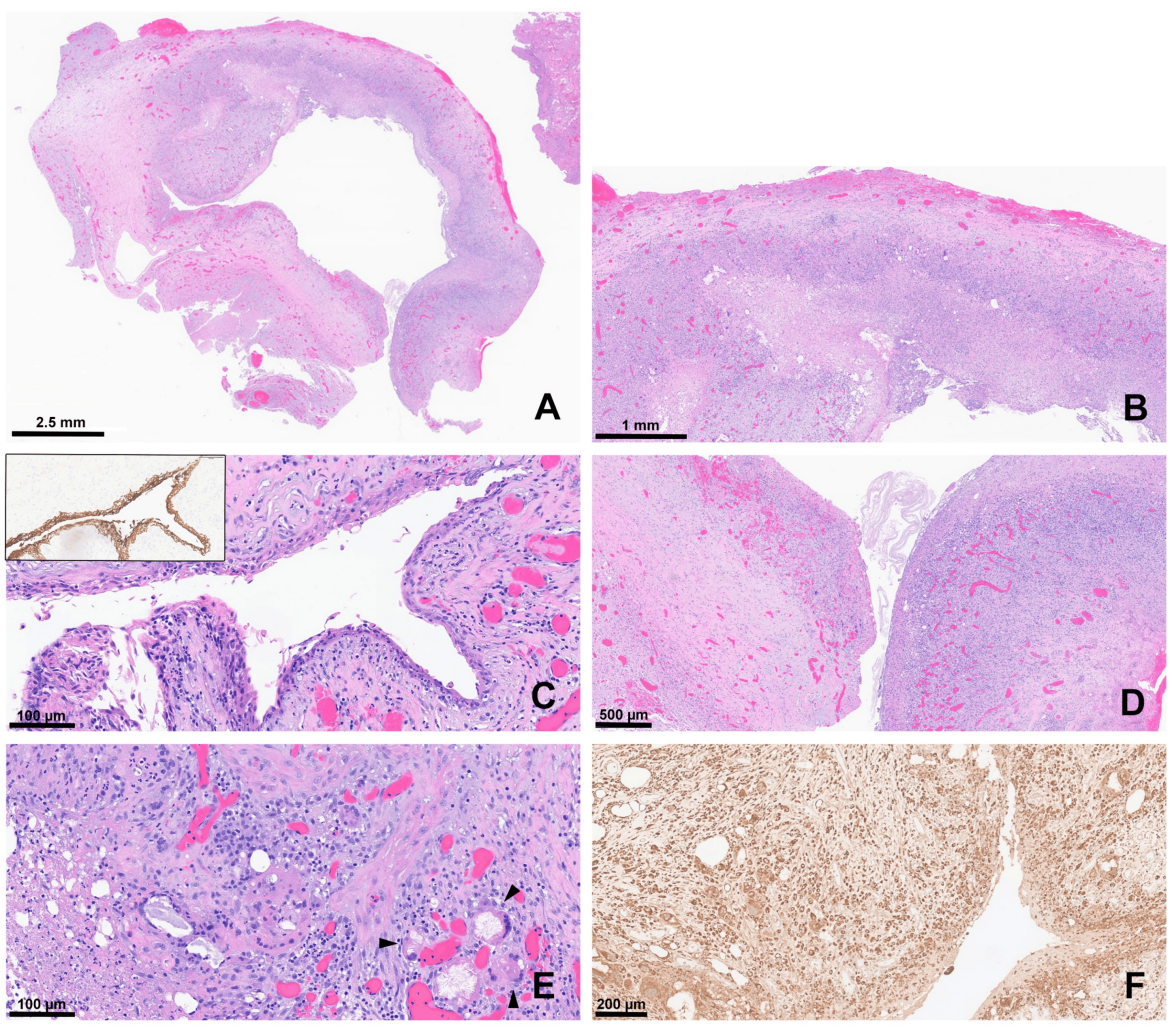
Surgical pathology specimen Ruptured epidermoid cyst: Sections reveal a large multiloculated cystic lesion with a thick, hyalinized, and vascular wall (A). The cyst wall is heavily denuded and shows areas of necrosis and extensive granulation tissue formation (B). Keratinized squamous epithelial lining focally lines the inner cyst wall (C) and is highlighted by keratin immunostain (inset: CKAE1/AE3). Strands and bundles of flaky keratin are seen within the cyst (D). Multinucleated giant cells, many filled with cholesterol clefts (arrowheads), are interspersed with sheets of macrophages (E). CD68 immunostain highlights the abundant macrophages throughout (F). CKAE1/AE3: Cytokeratin AE1/AE3; CD68: Cluster of differentiation 68

Following the recommendations agreed upon at a multidisciplinary tumor board discussion, the patient underwent a right craniotomy for biopsy and resection of the mass. Intraoperatively, the lesion was noted to have a rubbery and firm capsule. Debulking was performed to facilitate adequate resection and delineation of the brain-capsule interface. Upon opening the capsule, a dark fluid with motor oil-like viscosity was aspirated from inside the lesion. The frozen specimen was not consistent with a neoplastic process. Subsequent histological examination demonstrated a partially complex cystic lesion with a thickened, hyalinized wall and a patchy lining of keratinized squamous epithelium. Flaky keratin debris was identified within the cystic spaces. There was also marked acute and chronic inflammation, necrosis, and a foreign body-like reaction (abundant histiocytes and multinucleated giant cells) without evidence of malignancy, consistent with a ruptured EC (Figure 3).

Differential diagnostic considerations included dermoid cyst (no hair, hair follicles, or minor salivary glands), mature teratoma (no other elements identified), craniopharyngioma (no wet keratin or stellate reticulum), and Rathke cleft cyst. However, these diagnoses were deemed less likely. Additional immunohistochemistry and special stain preparations (Table 1) were performed, confirming the diagnosis of ruptured EC.

**Table 1:** Immunohistochemistry and special stains preformed on the surgical specimen CD68: Cluster of differentiation 68; CKAE1/3: Cytokeratin AE1/AE3; GFAP: Glial fibrillary acidic protein; GMS: Grocott methenamine silver; NeuN: Neuronal nuclei; SALL4: Spalt-like transcription factor 4; CCRCC: Clear cell renal cell carcinoma

Biomarker or stain	Significance	Result
CD68	Stain for macrophages/monocytes	Immunoreactive in the histiocytes and giant cells
CKAE1/3	Stain for epithelial tissue; useful for CCRCC and other malignant lesions	Immunoreactive in the squamous cell lining of the cyst
GFAP	Stain to determine if the tumor is glial in origin	Immunoreactive in gliotic brain tissue surrounding the lesion, but not immunoreactive in lesional cells
GMS	Stain to identify fungi	Negative for fungal organisms
NeuN	Mature neuronal cell marker	Not immunoreactive in lesional cells; high background and nonspecific staining
SALL4	Non-teratoma germ cell tumor maker	Not immunoreactive in lesional cells

**Figure 4:**
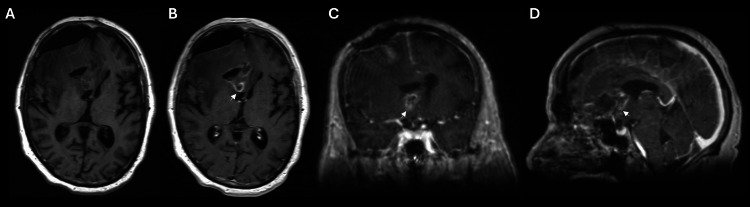
Post-operative MRI brain Post-operative MRI of the brain with axial T1 unenhanced image (A) demonstrates expected post-operative changes in the right frontal region, including expected pneumocephalus. The T1 post-contrast axial (B), coronal (C), and sagittal (D) images demonstrate a small amount of peripherally enhancing residual cystic lesion deep to the surgical site, extending into the remodeled and enlarged cribriform plate (arrowheads). Remodeling of the cribriform plate without destructive changes would suggest a relatively nonaggressive lesion, consistent with the pathologic diagnosis of epidermoid cyst. Mild leftward deviation of the right hemisphere is also present. MRI: Magnetic resonance imaging

The post-operative MRI demonstrated a small amount of residual cyst material at the medial base of the right frontal region, extending into the enlarged, remodeled cribriform plate (Figure 4).

The patient recovered from surgery with improved clinical neurological status. He was discharged to an acute inpatient rehabilitation facility. The patient progressed well clinically and functionally until postoperative day 18, when he woke with left-sided weakness and appeared to be more confused and somnolent to facility staff. Concurrently, he was noted to be febrile, with a temperature of 102.3°F. A stat CT demonstrated worsening vasogenic edema with increased midline shift. An MRI, with and without contrast, demonstrated peripheral enhancement within the surgical cavity. Additionally, central T1 hyperintensity was partially bright on diffusion-weighted imaging, suspicious for purulent material and raising concern for an infectious process. Analysis of cerebral spinal fluid (CSF) from a lumbar puncture was significant for leukocytosis with neutrophilia; however, gram stain and a viral meningitis panel were both negative. Initially, a diagnosis of Mollaret’s meningitis was favored. Four days after the lumbar puncture, the patient was noted to have purulent drainage from his cranial incision and was subsequently taken to the operating room for a wound washout. Operative cultures revealed methicillin-resistant *Staphylococcus aureus*. Postoperatively, the patient recovered again to his neurologic baseline. After the removal of his cranial drain, the patient was again discharged to a rehabilitation facility. 

## Discussion

Intracranial ECs are benign congenital lesions that develop from ectodermal remnants due to incomplete cleavage of the neural ectoderm from the cutaneous ectoderm during neural tube closure in the third to fifth weeks of embryogenesis [2,4,8]. This results in the entrapment of epithelial tissue within the neural tube, leading to the formation of ectodermal inclusion cysts [8]. The cyst capsule is composed of mature keratinized squamous epithelium, and the enlargement of the cyst is attributed to the accumulation of breakdown products from desquamating cells of the cyst wall. The accumulation of these breakdown products, mainly keratin and cholesterol, within the cyst lumen results in slow, linear enlargement of the cyst [8,9]. Due to their slow growth, these lesions typically have an insidious onset, as the brain has time to adapt and accommodate despite their often significant size and mass effect at presentation.

The case presented here is particularly notable due to several uncommon features that contributed to diagnostic challenges and additional surgical considerations.

Size and location 

The size and location of the cyst were atypical. The cyst was located in the frontal region near the midline, with extension into the cribriform plate. Very few ECs reach or exceed 5 cm in any one dimension and are referred to as giant epidermoid cysts (GECs) [8]. According to one recent study, only 26 cases meeting the criteria for GECs have been reported in the literature [8]. Regarding location, 90% of intracranial ECs are intradural, and almost all are extra-axial [2]. It is estimated that approximately 50% of intracranial ECs are found in the cerebellopontine cisterns, and 10-15% occupy suprasellar spaces or the middle cranial fossa (Sylvian fissure) [2,7]. Less common locations include the cerebral ventricles, mainly the fourth ventricle, and very rarely intraparenchymal [2].

Age at presentation 

The patient's advanced age at presentation contrasts with the typical age range for intracranial ECs. A case series from Pop et al. estimates the median age of diagnosis to be 40.4 years old [6]. Another case series by Gormley et al. reports an average age at presentation of 35 years old [10]. Generally, ECs are predicted to become symptomatic during the third to fifth decade of life [3,5]. This patient presented at 69 years old, with symptoms secondary to local mass effect and meningeal irritation from cyst rupture.

Radiographic features

Imaging studies play a critical role in characterizing ECs. On non-contrast head CT, ECs appear as well-defined CSF-like masses due to the combination of high cellular debris and cholesterol content, lowering the density of ECs to approximately 0 Hounsfield units [2,7]. On MRI, ECs are typically isointense to CSF on both T1- and T2-weighted images [2,7]. Most do not enhance, although minimal rim enhancement occurs in approximately 25% of cases [2,11]. The most defining radiographic feature is hyperintensity on fluid-attenuated inversion recovery (FLAIR) and diffusion-weighted sequences (i.e., restricted diffusion) [2,7,11].

The cyst in this case exhibited several atypical radiographic features on non-contrast head CT. For example, while it did demonstrate hypoattenuation, this attenuation was mixed and included fat attenuation with a density of approximately -29 Hounsfield units. Furthermore, the cyst contained minimal hyperdensity, which is a recognized but relatively rare feature of ECs [2,11]. Rather, these findings suggested a differential diagnosis of a dermoid cyst, which characteristically resembles fat (not CSF) on CT and routinely contains hyperdense plaques of calcification within the cyst capsule [2]. Finally, the considerable amount of vasogenic edema associated with this patient’s cyst is highly unusual for ECs. We speculated that the presence of this edema suggested a secondary inflammatory or reactive process triggered by cyst rupture. Again, we postulated a likely ruptured dermoid cyst, as these have a higher tendency toward rupture compared to an EC. The presence of vasogenic edema also prompted the consideration of differential diagnoses such as malignant transformation or an infectious process.

On MRI, the cyst displayed unconventional complex signal characteristics, including heterogeneous T1 and T2 signal intensities, as well as a fluid-fluid level in the posterior aspect. Given these radiographic features, differential considerations included a dermoid cyst with secondary infection and resultant brain abscess, teratoma, and atypical meningioma. The lesion did not invade or destroy bone, nor did it involve the sinonasal mucosa, so more sinister etiologies, such as squamous cell carcinoma and esthesioneuroblastoma, were excluded. Meningioma was in the differential but less favored, as there was no dural tail present. Definitive diagnosis required histopathological examination of intraoperative samples, which confirmed the diagnosis of a ruptured EC. Suspicions of a teratoma or mixed germ cell tumor in the differential were effectively ruled out through additional immunohistochemical studies (Table 1).

Spontaneous rupture

Spontaneous rupture of an EC is a rare yet potentially fatal presentation due to the drainage of the EC fluid into the subarachnoid space, causing chemical meningitis [12-14]. Chemical meningitis is a form of aseptic meningitis that is diagnosed by negative spinal fluid Gram stain and cultures [14]. The ensuing inflammatory reaction has the potential to trigger vasospasm, with subsequent infarction or even death [2]. The unusual presentation of this patient, with rupture and subsequent aseptic meningitis, underscores the variability in clinical course and potential complications associated with these lesions.

ECs lack radio-sensitivity, rendering complete surgical excision the mainstay of treatment [9,15]. These cysts tend to surround and envelope, rather than displace, nerves and vessels [15,16]. Although it would prevent recurrence, radical resection is not recommended when the cyst capsule is adherent to surrounding neurological and vascular structures [16]. In such instances, the suggested approach is conservative, given the benign histological nature of these lesions, with prioritization of sparing structures whose damage would negatively impact the patient’s quality of life [16]. In the present case, near-total resection was performed, as complete removal was technically challenging and risked causing neurological injury to the patient. 

Following resection of the cyst, the patient's clinical course was again complicated by chemical meningitis. In contrast to spontaneous rupture, chemical meningitis is a well-described postoperative complication of ECs, occurring in 40% of patients undergoing subtotal resection [9,17]. The risk can be minimized by administering systemic corticosteroids perioperatively [17]. Prompt initiation of systemic corticosteroids led to clinical improvement in this patient, highlighting the importance of postoperative monitoring and early intervention in managing complications.

Mollaret's meningitis 

Although an uncommon cause of recurrent aseptic meningitis, ECs have been described in association with Mollaret’s meningitis [3,18]. A commonly described scenario is one in which patients present with recurrent bouts of aseptic meningitis, and then it is discovered that the patient has an undiagnosed EC [3,19]. In our case, the patient developed signs of Mollaret’s meningitis but was ultimately found to have a methicillin-resistant *Staphylococcus aureus* wound infection despite sterile CSF on lumbar puncture. Given that the patient displayed signs of worsening edema after surgery in conjunction with postoperative fevers, high clinical suspicion for a postoperative wound infection should remain, despite the association with Mollaret’s meningitis in the setting of residual mass.

## Conclusions

This case report underscores the diagnostic and therapeutic challenges associated with intracranial ECs, particularly when presenting in an unusual location and with atypical complications, such as cyst rupture and subsequent infection. Comprehensive evaluation, including advanced imaging modalities and histopathological analysis, is essential for accurate diagnosis and optimal surgical management. Further research is needed to better understand the pathogenesis and natural history of these rare lesions and to refine treatment strategies aimed at improving patient outcomes and reducing postoperative complications.
